# Radiolabeled LHRH and FSH Analogues as Cancer Theranostic Agents: A Systematic Review

**DOI:** 10.3390/jcm14217811

**Published:** 2025-11-03

**Authors:** Anna Giorgio, Michela Varani, Chiara Lauri, Valeria Bentivoglio, Pallavi Nayak

**Affiliations:** Nuclear Medicine Unit, Department of Medical-Surgical Sciences and of Translational Medicine, Faculty of Medicine and Psychology, “Sapienza” University of Rome, 00189 Rome, Italy; michela.varani@uniroma1.it (M.V.); chiara.lauri@uniroma1.it (C.L.); valeria.bentivoglio@uniroma1.it (V.B.); pallavi.nayak@uniroma1.it (P.N.)

**Keywords:** radiopharmaceuticals, LHRH analogue, FSH analogue, nanotechnology, cancer

## Abstract

**Background:** G-protein-coupled receptors (GPCRs) play pivotal roles in tumor growth and progression. Among these, the luteinizing hormone-releasing hormone receptor (LHRH-R) and follicle-stimulating hormone receptor (FSH-R) represent promising translational targets, unlike luteinizing hormone receptors (LH-Rs). Indeed, both LHRH-R and FSH-R are selectively expressed in various cancers and their vasculature, offering opportunities for receptor-mediated imaging and therapy. **Objectives:** This systematic review aims to evaluate radiolabeled LHRH- and FSH-derivative biomolecules, including peptides, monoclonal antibodies and nanocarriers, for their applications in cancer diagnosis and treatment. **Methods:** The systematic review was conducted following PRISMA 2020 guidelines. A systematic search of PubMed, Scopus and Web of Science was conducted for studies published between 2005 and 2025. A total of 248 records were identified, and 156 articles were screened after removing duplicate records. Two authors independently selected eligible studies. Quality of evidence was assessed by the *Quality Assessment of Diagnostic Accuracy Studies* (QUADAS) approach. **Results:** A total of 25 studies met the inclusion criteria and were included in the final review. Radiolabeled LHRH and FSH derivatives showed receptor-specific tumor localization in both preclinical and clinical applications. FSH-R expression in tumor blood vessels supports its potential as a biomarker for early cancer diagnosis. FSHβ-derived peptides exhibit improved pharmacokinetics compared to monoclonal antibodies in PET imaging. LHRH analogues, particularly D-Lys^6^-modified peptides, proved effective for SPECT, PET and therapeutic applications, particularly in breast and prostate cancer. The integration of radiolabeled LHRH and FSH derivatives with nanocarriers further enhanced probe stability and tumor targeting, increasing tumor accumulation and image contrast compared to free peptide. **Conclusions:** Radiopharmaceuticals targeting LHRH-R and FSH-R are promising tools for cancer imaging and treatment. Advances in nanotechnology enhance delivery precision and reduce systemic toxicity, thereby increasing its translational promise in oncology.

## 1. Introduction

G-protein-coupled receptors (GPCRs) are the largest and most common class of cell surface signaling receptors known to play essential roles in physiological activities, including tumor growth and metastasis. A broad range of ligands, including hormones, lipids, peptides and neurotransmitters, activate GPCRs, enabling these receptors to interact with G-proteins and activate multiple downstream signaling pathways. Integration of these complexes’ signaling networks produces numerous biochemical responses that drive diverse pathophysiological processes, including cancer development. In the GPCR family, the luteinizing hormone-releasing hormone receptor (LHRH-R) and follicle-stimulating hormone receptor (FSH-R) offer strong translational potential [1].

**Figure 1:**
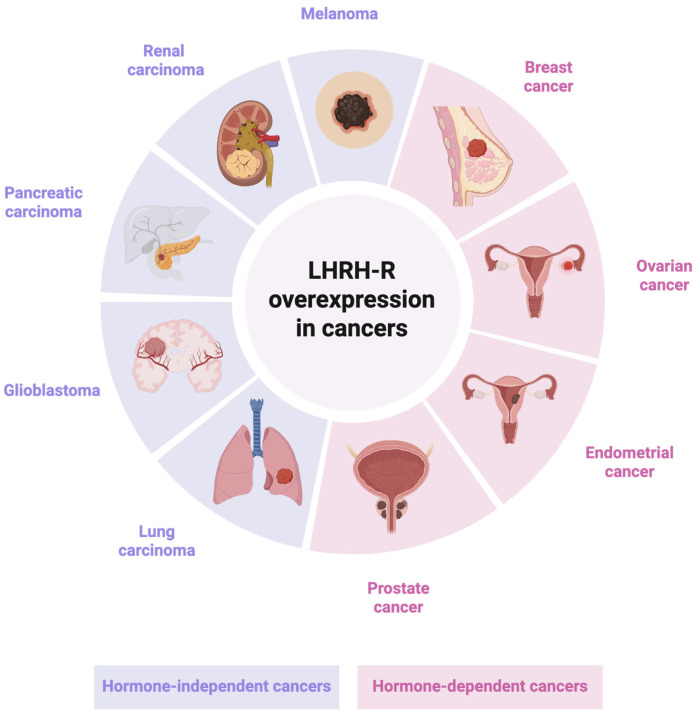
LHRH overexpression in hormone-dependent/independent cancers (created using BioRender.com).

LHRH, also known as gonadotropin-releasing hormone (GnRH), is a hypothalamic decapeptide (sequence: pGlu-His-Trp-Ser-Tyr-Gly-Leu-Arg-Pro-Gly-NH_2_) that plays a pivotal role in the regulation of reproductive physiology. It stimulates the pituitary gland to release LH and FSH, which control the production of gonadal sex steroids in both males and females [2,3]. In addition to its well-established role in the hypothalamic–pituitary–gonadal axis, expression of extra-pituitary LHRH-R (or GnRH-R) has been observed in several tissues and malignancies, including cancer cells [4]. Although its biological role in tumors remains unclear, evidence suggests that LHRH peptides may act as local regulators of tumor growth [5]. Early studies primarily linked LHRH-R overexpression to hormone-dependent cancers [6], such as breast [7], endometrial [8], ovarian [9] and prostate cancers [10] (Figure 1). However, subsequent findings have demonstrated variable receptor expression in several hormone-independent cancers, including pancreatic [11], renal [12] and lung carcinomas [13], as well as melanoma [14] and glioblastoma [15] (Figure 1).

Notably, LHRH-R expression in these cancers is often significantly higher than in corresponding normal tissues, including those of reproductive origin. Clinical investigations have also shown that treatment with LHRH agonists does not exert substantial therapeutic effects in cancers characterized by LHRH-R overexpression [16]. Nonetheless, the selective expression of these receptors in tumors highlights the potential of LHRH targeting peptides and their derivatives as drug-delivery systems, enabling targeted therapy while sparing healthy tissues. As highlighted by Li and colleagues, various LHRH-targeted drug delivery approaches are currently under investigation for oncological applications [17,18,19].

Similarly, FSH-R may also be considered a promising biomarker for targeted therapy and diagnosis in aggressive tumors [20]. FSH-Rs are usually expressed by cells of the gonads and are absent in other normal tissues. They are mainly overexpressed by ovarian and prostate cancer cells [20,21].

**Figure 2:**
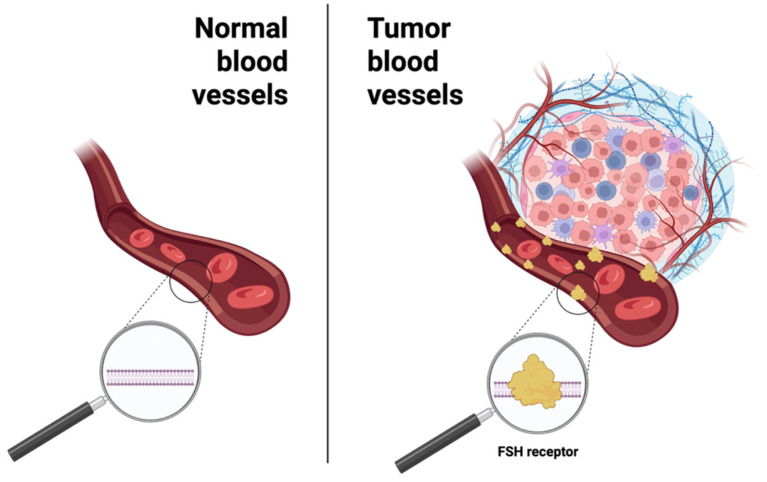
FSH-R is a highly selective TBV marker for primary and metastatic solid tumors (created using BioRender.com).

In the 2010s, FSH-R raised renewed interest as a highly selective marker for tumor blood vessels (TBVs) in primary and metastatic solid cancers, detectable at very early stages of the disease (Figure 2).

Adjacent normal tissues and blood vessels in inflammatory lesions and in normal wound healing were uniformly negative for FSH-R [22,23]. Notably, FSH-R-positive (FSH-R+) TBVs are located at the boundary between tumoral and normal tissues, suggesting that FSH-R contributes to tumor initiation, progression and metastasis, consistent with its established role in regulating ovarian angiogenesis [21]. Additionally, FSH-R expression is not restricted to gonadal tumors [20]. Recently, it has been demonstrated that a wide range of human and murine cancer cells heterogeneously express FSH-R. To this end, many efforts have already been made to design new tools for noninvasive imaging of FSH-R.

Since LHRH-R and FSH-R are overexpressed only in cancer cells [16,17,24,25] and absent in normal tissues, they can be considered a selective target useful to minimize off-target radiation exposure both in imaging and therapy.

Recent advances in the development of radiolabeled LHRH and FSH biomolecules, such as antibodies and peptides and integration with nano-platforms, have been widely used for single-photon emission computed tomography (SPECT, using ^99m^Tc and ^111^In) and positron emission tomography (PET, using ^68^Ga and ^18^F) imaging, with a smaller number evaluated also for therapeutic use (^177^Lu).

Radiolabeled monoclonal antibodies (mAbs) are evaluated for their ability to bind with high specificity and affinity to defined molecular structures, which makes them particularly suitable for both therapeutic and diagnostic applications. The use of monoclonal antibodies in nuclear medicine offers several advantages but also presents limitations. Radiolabeled mAbs offer excellent target selectivity and can provide insights into tumor heterogeneity, receptor density and disease progression over time. This precision enables personalized diagnostic strategies and potentially aids in treatment planning. However, the relatively large size of mAbs leads to slower pharmacokinetics, prolonged circulation times and sometimes suboptimal tumor penetration. Additionally, the use of long-lived radionuclides may increase radiation burden, while antibody production itself can be costly and technically demanding.

Radiolabeled peptides have emerged as a versatile class of molecular probes in nuclear medicine, with applications in both diagnostic imaging and targeted radionuclide therapy. Their relatively small size, compared to monoclonal antibodies, facilitates rapid tissue penetration and favorable pharmacokinetics, resulting in a high target-to-background contrast within a shorter time frame. Moreover, their small size also ensures rapid clearance from the bloodstream.

Peptides can be chemically modified with relative ease, allowing optimization of pharmacokinetics, enzymatic stability and receptor binding. However, sometimes their rapid renal clearance may lead to insufficient tumor retention, reducing sensitivity in imaging and efficacy in therapy. Peptides generally exhibit lower absolute binding specificity compared to whole antibodies, which may increase background signal in specific settings. Their relatively short in vivo half-life may necessitate the use of radionuclides with matching physical properties, limiting flexibility in probe design.

**Figure 3:**
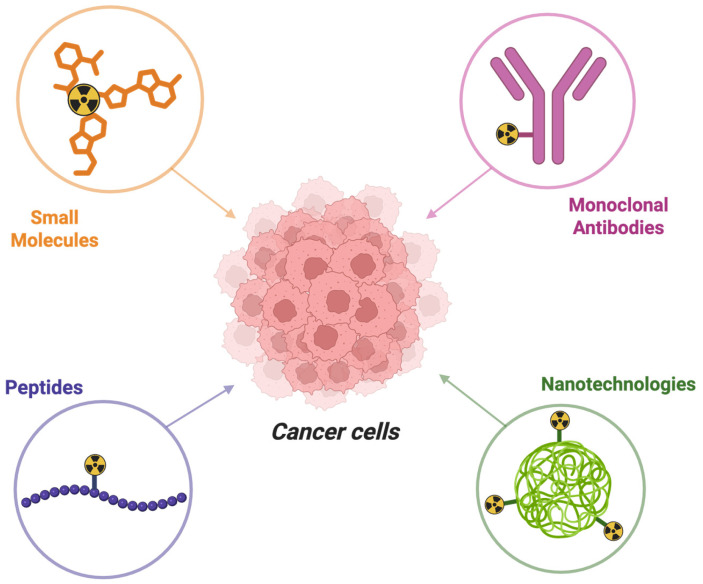
Different radiopharmaceuticals useful in nuclear medicine: radiolabeled small molecules, peptides, monoclonal antibodies and nanotechnologies (created using BioRender.com).

Furthermore, nanotechnology is increasingly applied in nuclear medicine to enhance the delivery and efficacy of radiopharmaceuticals. Gold nanoparticles, BSA nanoclusters and also solid-lipid nanocarriers are just some of the examples of nanotechnologies currently in use which have been identified for applications in targeted imaging of tumors overexpressing specific receptors (Figure 3).

This systematic review compiles the studies reported over the past 20 years on radiolabeled LHRH and FSH biomolecules for cancer management. We include radiolabeling chemistry and characterization of peptide and antibody conjugates, in vitro and in vivo biodistribution and pharmacokinetics, clinical investigations and integrations with nanotechnology platforms.

Our objective is to integrate the reported studies to evaluate LHRH/FSH targeting for consistent radiolabeling and receptor-specific localization in tumors and their vasculature, thereby supporting present and future theranostic applications.

## 2. Materials and Methods

### 2.1. Search Strategy

The published studies were systematically collected using databases such as PubMed, Scopus and Web of Science. A search algorithm based on the combined terms “((“Follicle stimulating hormone” OR FSH) AND “radiolabeling” AND “cancer”)”, “((“Luteinizing hormone releasing hormone” OR LHRH) AND “radiolabeling” AND “cancer”)”, “((“Gonadotropin releasing hormone” OR GnRH) AND “radiolabeling” AND “cancer”)” was employed. Reference lists of included studies and relevant reviews were also screened to identify additional records.

Other filters were applied such as “Research Articles” and “Publication year from 2005 to 2025”. In PubMed a database-specific date filter was used, selecting papers from “1 January 2005” to “30 September 2025”.

Only original research papers published in English were considered, following the PRISMA 2020 guidelines [26,27].

Quality control was ensured by implementing the PRISMA checklist (Supplementary Table S1). Two authors independently critically assessed the overall quality of the selected studies. Only studies using radionuclides for nuclear medicine applications were included.

### 2.2. Inclusion and Exclusion Criteria

Literature studies published up to September 2025 were assessed. The bibliography of the research articles was manually searched to assess further relevant articles. Full-text articles were evaluated, and preclinical results were considered for inclusion and analysis. Letters to the editor, supplementary commentaries, book chapters, review articles and non-English publications were excluded from the analysis.

### 2.3. Data Extraction and Management

Two authors independently screened full-text manuscripts for eligibility, summarizing essential data. Risk assessment of any potential bias and data collection were performed using a standardized questionnaire reported in Appendix A (Table A1). The questionnaire was adapted to the studies included in this systematic review.

### 2.4. Assessment of Risk Bias in Included Studies

All included studies were evaluated by using the *Quality Assessment of Diagnostic Accuracy Studies* (QUADAS) approach for any potential source of bias and variation [27]. To obtain all this information, each study was analyzed individually.

The following variables were analyzed: (1) animal selection bias (animal origin, animal model and sex distribution), (2) animal variation (sex distribution and disease severity), (3) index test bias (bacterium origin, bacterium number, conjugation chelator-biomolecule, radiochemical purity and extra in vitro/in vivo/ex-vivo studies), (4) index test variation (observer variation, availability of experimental information, radiopharmaceutical purity and specific activity and test execution), (5) reference standard bias (inappropriate or inconsistent reference standard and incorporation bias), (6) reference standard variation (definition of a control model) and (7) flow and timing bias (radiopharmaceutical administration time, imaging time bias, uninterpretable results and study flow).

## 3. Results and Discussion

### 3.1. Data Synthesis

**Figure 4:**
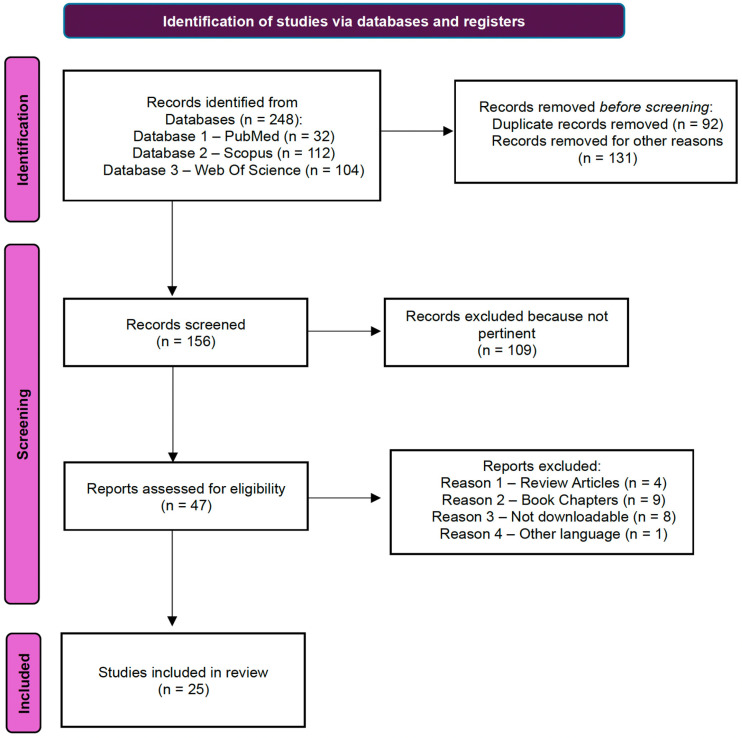
A flowchart illustrating the search for eligible studies.

Studies for this systematic review were selected by two authors. In Figure 4 a flowchart illustrating the search for eligible studies is reported. Starting from 248 papers identified on three different databases (32 on PubMed, 112 on Scopus and 104 on Web of Science), duplicate records were removed (*n* = 92). Of the 156 remaining works, 109 were excluded because they were not pertinent to our criteria (research articles regarding FSH- and LHRH-derivative radiopharmaceuticals useful in nuclear medicine). Following this procedure, 47 potential studies were identified. After excluding non-review articles (*n* = 4), book chapters (*n* = 9), non-downloadable (*n* = 8) and other language (*n* = 1) articles, only 25 published articles were selected for our study. A summary of each paper’s results is reported in the following paragraphs.

**Figure 5:**
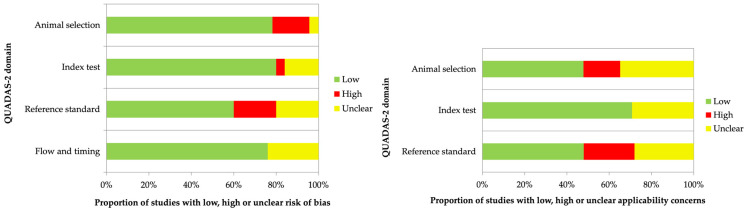
Graphical representation of frequencies of biases in analyzed papers by QUADAS 2.

In Table 1 and Figure 5, QUADAS analysis results are reported. To facilitate the analysis, the studies were divided into analyses of radiopharmaceuticals derived from FSH and LHRH, and for the latter, they were further divided into SPECT, PET, therapy and other applications. Selected papers appear to be heterogeneous as they involve only in vitro and in vivo studies.

### 3.2. Radiolabeled Biomolecule Targeting FSH-R

FSH-R has the potential to serve as a valuable biomarker in oncology, facilitating early cancer detection and contributing predictive value to personalized medicine. In 2005, Fan and Hendrickson solved the first crystallographic structure of the FSH hormone in a complex with the extracellular domain of the FSH-R [53]. FSH is a heterodimeric glycoprotein consisting of two distinct subunits, α and β, with the latter conferring biological activity and FSH-R specificity [54]. The interaction between the glycoprotein hormones and their corresponding receptors is highly selective: the FSH-FSH-R structure allows us to understand in detail the molecular requirements underlying the interaction specificity [53] and paves the way for the design of highly specific and selective FSH derivatives. Some of them are based on monoclonal antibodies (mAbs) or peptides derived from the human FSHβ portion. Molecular imaging with radionuclides, such as PET, provides a powerful tool to visualize and monitor dynamic biochemical processes and the distribution of specific molecular targets in vivo. To improve the tumor localization and the sustained detection of FSH-R expression, researchers have applied PET imaging using different radiopharmaceuticals using both monoclonal antibodies and peptides.

#### 3.2.1. Radiolabeled Monoclonal Antibodies Against FSH-R

In this context, a monoclonal antibody (FSH-R mAb) against FSH-R has already been used in a nuclear medicine (Table 2).

In 2015, Hong and coworkers performed functionalization with a chelator agent and radiolabeling with ^64^Cu for PET imaging of FSH-R in various tumor models [28]. They developed ^64^Cu-NOTA−FSH-R mAbs, an immune-PET tracer for FSH-R. Since FSH-R is selectively expressed in the vasculature of a wide range of solid tumors but largely absent from most normal tissues, the authors aimed to establish its broad applicability as a molecular imaging target. In this study, in vivo PET/CT in nude mice bearing CAOV-3 (FSH-R+) ovarian, SKOV-3 (FSH-R-) ovarian, MDA-MB-231 breast and PC-3 prostate xenografts showed robust, time-dependent uptake. Histology studies confirmed widespread microvascular FSH-R across models and notable tumor-cell FSH-R in CAOV-3, PC-3 and MDA-MB-231; the authors note that endothelial versus tumor-cell contributions could not be fully disentangled, and in vivo blocking was not performed due to limited antibodies. Overall, ^64^Cu-NOTA-FSH-R mAbs enabled specific, quantitative imaging of FSH-R across multiple tumor types, supporting FSH-R as a broadly applicable target for non-invasive cancer imaging and potential theranostics.

One year later, Yang et al. engineered a tumor-vasculature-targeted nano-graphene oxide (GO) platform [29]. GO nanosheets were initially functionalized with branched PEG, and after they were attached to NOTA and FSH-R mAbs and radiolabeled with ^64^Cu. Graphene derivatives are considered attractive drug-delivery systems. In this study, the authors demonstrated efficient metastatic tumor targeting of GO conjugates in vivo, in a murine model of lung metastasis for breast cancer. In vitro, fluorescein-GO-FSH-R-mAbs bound MDA-MB-231 cells (FSH-R+) far more strongly than non-targeted GO, with negligible uptake in SKOV-3 cells (FSH-R-); antibody denaturation abrogated binding, confirming specificity. In an experimental lung-metastasis model (female nude mice injected intravenous with cbgLuc-MDA-MB-231), serial PET at increasing times post-injection showed rapid and sustained uptake of ^64^Cu-NOTA-GO-FSH-R-mAbs in metastatic nodules, significantly exceeding both non-targeted ^64^Cu-NOTA-GO and a denatured-antibody control. Biodistribution indicated predominant hepatobiliary clearance and low muscle background, yielding high-contrast detection of micro-metastases; histology localized GO-FSH-R mAbs to tumor vasculature and confirmed liver/spleen capture. As a drug carrier, GO-FSH-R mAbs demonstrated a high doxorubicin loading capacity and enhanced delivery to metastatic sites, as confirmed by fluorescence imaging, supporting this system as a dual diagnostic–therapeutic platform for early metastasis detection and targeted delivery.

So, as can be understood from these studies, antibodies represent an excellent alternative in PET imaging. Despite these results, there are limits due to their nature, such as long circulating half-life (t_1/2_) and nonspecific accumulation, with consequent poor tumor-to-blood and tumor-to-organ ratios. Conversely, different peptides have been tested with promising results, and the most successful turned out to be FSHβ33-53, a linear peptide deriving from the human FSHβ portion [54].

In the QUADAS analysis, no sources of bias were reported for these two studies.

#### 3.2.2. Radiolabeled FSHβ-Derivative Peptides in Cancer Imaging

FSHβ33-53 has been investigated since the early 90s by Santa Coloma and coworkers for ovarian cancer targeting [54,55]. The amino acid residues of FSHβ that interact with the receptor are found both in the loop containing the sequence FSHβ 95–100 and in the region of the loop comprising residues 38–48, as reported by Jiang and colleagues [56].

FSHβ33–53 has recently gained interest as a development for FSH-R-selective drugs and radiopharmaceuticals for cancer imaging [30,31,32,33] and therapy [28,57]. It demonstrates excellent in vivo stability and FSH-R specificity but shows suboptimal pharmacokinetics and tumor-to-kidney ratios [30,31,32]. These limitations are attributed to dimerization at physiological pH via intermolecular disulfide bonds between cysteine residues on the monomers [55]. In general, peptide dimerization may reduce its biological efficacy by altering conformation and solubility, decreasing receptor specificity and bioavailability, limiting tumor penetration and leading to suboptimal tumor-to-kidney ratios.

In this context, four different research articles reporting radiolabeled FSHβ-derivative peptides have been published (Table 3). The first one was published in 2014 by Xu and coworkers, who synthesized and characterized ^18^F-Al-NOTA-MAL-FSH1, a novel ^18^F-labeled FSH-R probe for tumor PET imaging [30]. In vivo evaluation was performed in nude mice bearing FSH-R-positive and FSH-R-negative PC-3 xenograft tumors. PET imaging demonstrated clear visualization of FSH-R-positive tumors, with significantly higher tracer uptake compared to controls and favorable tumor-to-muscle ratios. The probe showed rapid tumor accumulation evident within 30 min post-injection and relatively low background activity, except for renal clearance. Receptor-blocking experiments further validated the specificity of tumor uptake. This peptide has favorable pharmacokinetics, specific tumor targeting, rapid blood clearance and predominantly renal excretion. ^18^F-Al-NOTA-MAL-FSH1 represents a potential and promising radiotracer for the non-invasive visualization of FSH-R-positive tumors in vivo.

In 2016, Zhu and coworkers modified the FSH-R antagonist peptide sequence with a hydrophilic linker (^18^F-Al-NOTA-MAL-FSH2) to improve the pharmacokinetic properties and reduce nonspecific uptake in non-target tissue [31]. This radiolabeled peptide remains stable in PBS and human serum. In vitro assays confirmed high binding affinity and specificity for FSH-R. PET imaging of nude mice bearing FSH-R-positive PC-3 xenografts demonstrated pronounced tumor uptake and clear visualization against a low background. In conclusion, ^18^F-Al-NOTA-MAL-FSH2 showed better pharmacokinetics than the previously reported ^18^F-Al-NOTA-MAL-FSH1. A favorable preclinical study revealed that the incorporation of a hydrophilic linker enhances the imaging performance. ^18^F-Al-NOTA-MAL-FSH2 appears to be a promising candidate for FSH-R-positive tumor imaging.

In addition to ^18^F, ^68^Ga is currently a promising PET radionuclide due to its availability, nearly quantitative reaction and short physical half-life. In 2017, Pan and coworkers developed a ^68^Ga labeled FSH1 peptide for FSH-R imaging [32]. In vitro studies and microPET imaging were performed in the PC-3 prostate tumor model. [^68^Ga]Ga-NOTA-MAL-FSH1 was stable in PBS and human serum for at least 2 h. PET imaging revealed that the PC-3 xenografts were clearly visualized, with specific tumor uptakes and good tumor-to-background contrast, with rapid renal excretion and minimal nonspecific accumulation in other organs. Biodistribution studies confirmed these findings, demonstrating high tumor-to-muscle ratios and receptor-mediated specificity confirmed by blocking experiments. Preclinical data obtained in this paper indicate that this radiopharmaceutical is promising for noninvasive visualization of FSH-R expression in vivo, even in other cancer types.

In the QUADAS analysis, the main source of bias was related to the animal sex distribution and inconsistent reference standards and definitions of control models being applied.

In 2022, Pan and colleagues optimized the performance of [^68^Ga]Ga-NOTAMAL-FSH1 in a prostate cancer model by co-administration of aprotinin [33]. Aprotinin is a competitive, reversible, heat- and acid-stable inhibitor of proteolytic and esterolytic activities. To improve the in vivo stability and tumor uptake of peptide-radiopharmaceuticals, the authors modified the amino acid sequence, resulting in [^68^Ga]Ga-NOTAMAL-FSH4, and evaluated it with aprotinin. In vivo studies in nude mice bearing PC-3 prostate cancer xenografts demonstrated that co-injection with aprotinin further increased tumor uptake and tumor-to-background contrast while maintaining low nonspecific accumulation in non-target tissues. Biodistribution analyses confirmed enhanced tumor retention and favorable tumor-to-muscle ratios without affecting renal clearance. All these findings indicate that the sequence modification combined with protease inhibition effectively improves the performance of the ^68^Ga labeled peptide to benefit future clinical applications.

Molecular PET imaging of FSH-R is beneficial for cancer prognosis, and the findings related to the FSHβ33–53 peptide confirmed its receptor selectivity and affirmed its relevance in nuclear medicine imaging applications.

### 3.3. Radiolabeled LHRH Derivative Peptides

A significant number of tumors overexpresses the gonadotropin-releasing hormone type 1 receptor (GnRH1 receptor), which offers a promising molecular target for both diagnostic imaging by PET or SPECT and radionuclide therapy. More generally, GnRH receptors are overexpressed in various hormone-dependent (and independent) malignancies, including prostate, breast and ovarian cancers, making them attractive targets in nuclear medicine. Several preclinical studies have explored radiolabeled analogues of GnRH (or LHRH) peptides as agents for receptor-targeted nuclear medicine applications, and the following works provide critical insights into their development and translational potential.

In the following paragraphs, a wide variety of radiolabeled biomolecules suitable for overexpressing LHRH cancer imaging, specifically for PET (^68^Ga, ^18^F), SPECT (^111^In, ^99m^Tc) and therapy (^177^Lu) applications, are reported. In addition, we also describe these biomolecules conjugated with novel nanotechnology, like nanoparticles and macroaggregates.

#### 3.3.1. Radiolabeled LHRH in SPECT Imaging Applications

Several LHRH derivative peptides radiolabeled with ^99m^Tc and ^111^In have been published (Table 4).

In 2019 Zoghi and coworkers investigated the synthesis, radiolabeling and preclinical evaluation of a novel ^111^In-labeled GnRH-I peptide-based radiopharmaceutical ([^111^In]-DOTA-TRP) designed for SPECT imaging of tumors overexpressing GnRH1 receptors [34]. The radiolabeled compound demonstrated excellent stability in vitro both in PBS and in human serum, with minimal degradation observed over 24 h. In vitro receptor binding assays confirmed that the radiolabeled peptide retained its affinity for GnRH-I receptors, indicating that the DOTA conjugation and radioisotope incorporation did not compromise its biological function. In biodistribution studies performed in normal and tumor-bearing mice (4T1-bearing mice), the tracer exhibited favorable pharmacokinetics with rapid clearance from the bloodstream, primarily through the renal pathway and low non-specific uptake in non-target tissues. Most importantly, the radiopharmaceuticals showed target-specific accumulation in tumors, with significantly higher uptake in receptor-positive tumors compared to background organs and muscle, leading to promising tumor-to-nontumor contrast ratios. Distinct ovarian uptake was also observed. In the end, authors presented [^111^In]-DOTA-TRP as a promising radiotracer candidate for GnRH receptor SPECT imaging. The results support further preclinical validation and provide a solid basis for future clinical translation, particularly in the context of ovarian and other GnRH receptor-rich tumors.

In 2011, Guo and coworkers developed three novel DOTA-conjugated peptides for breast cancer imaging [35]. The chelator was conjugated in different positions: to the epsilon or alpha amino group of D-lysine or the epsilon amino group of L-lysine via an Ahx linker, obtaining DOTA-Ahx-(D-Lys^6^-GnRH1), DOTA-Ahx-(D-Lys^6^-GnRH2) and DOTA-Ahx-(L-Lys^6^-GnRH3). Only the DOTA-Ahx-(D-Lys^6^-GnRH1) peptide maintained the nanomolar GnRH receptor binding affinity. Moreover, the biodistribution and tumor imaging properties of ^111^In-DOTA-Ahx-(D-Lys^6^-GnRH1) were evaluated in MDA-MB-231 human breast cancer-xenografted nude mice. These radiopharmaceuticals exhibited specific GnRH receptor binding, rapid tumor uptake and fast clearance through the urinary system. Tumor lesions were clearly visualized 1 h post-injection by SPECT/CT. Ultimately, authors demonstrated the impact of chelator position on the binding affinity for the receptor and the potential of the radiopharmaceuticals as a novel imaging probe for human breast cancer imaging.

Based on previous results, Guo and coworkers designed a new study in 2011 by radiolabeling (DOTA)-Ahx-(D-Lys^6^-GnRH1) with ^111^In [36]. The authors evaluated the tumor targeting and imaging properties of the novel radiopharmaceuticals for human prostate cancer, both in vitro and in vivo, in DU145 human prostate cancer-xenografted nude mice. The radiopharmaceuticals exhibited high binding affinity and specific internalization in GnRH receptor–positive cells, and in vivo biodistribution studies demonstrated rapid tumor uptake, fast clearance from blood and non-target tissues and clear tumor visualization on SPECT/CT images within 30 min of administration. Renal retention was observed due to the pharmacokinetic profile of the small peptide, but overall tumor-to-background ratios were favorable. The ability of ^111^In-DOTA-Ahx-(D-Lys^6^)-GnRH1 demonstrated its promise as a novel probe for the imaging of human prostate tumors.

In 2017, Xu and coworkers reported the design, synthesis and biological evaluation of novel ^111^In-labeled GnRH peptide analogues for prostate cancer imaging [37]. Three GnRH peptides were synthesized with different hydrocarbon linkers: ^111^In-DOTA-Aoc-D-Phe-(D-Lys^6^-GnRH), ^111^In-DOTA-βAla-D-Phe-(D-Lys^6^-GnRH) and ^111^In-DOTA-Aun-D-Phe-(D-Lys^6^-GnRH). The authors demonstrated that Aoc is better than βAla and Aun linkers in retaining strong receptor binding affinity. ^111^In-DOTA-Aoc-D-Phe-(D-Lys^6^-GnRH) shows faster tumor uptake and urinary clearance in DU145 human prostate cancer-xenografted nude mice. These findings highlighted the potential of this ^111^In-labeled GnRH peptide as a promising molecular probe for prostate cancer imaging by SPECT, supporting further optimization and translational research in this area.

Farahani et al. conducted a series of preclinical studies to evaluate a ^99m^Tc-labeled-DLys^6^-GnRH analogue for prostate cancer imaging [38,39]. In one report, the peptide was modified with aminobutyric acid (GABA) as linker (^99m^Tc-HYNIC-GABA-D-Lys^6^-GnRH) [38], while, in another report, amino hexanoic acid (Ahx) was used as a hydrocarbon linker to generate ^99m^Tc-HYNIC-Ahx-DLys6-GnRH [39]. Both the analogues showed the highest affinity for LN-CaP cells. Reported Kd values were ~89 nM (GABA-linker analogue) and ~42 nM (Ahx-linker analogue), indicating stronger binding affinity for the Ahx-containing derivative. In vivo biodistribution studies in LN-CaP xenografted nude mice revealed similar pharmacokinetic profiles for both tracers, but tumor uptake and imaging contrast differed. The GABA analogue achieved a tumor uptake of 1.72 %ID/g at one hour with a tumor-to-muscle ratio of 2.3. In contrast, the Ahx analogue reached a higher uptake of 3.67 %ID/g at one hour and a high tumor-to-muscle ratio of 4.14. SPECT imaging confirmed clear visualization of LN-CaP tumors within 1–2 h for both agents, with the Ahx variant producing sharper contrast. Collectively, these studies demonstrate that radiolabeled GnRH analogues reliably target GnRH receptor-positive prostate cancer.

In 2017, Hao and colleagues performed direct radiolabeling of ^99m^Tc with LHRH using a pre-thinning approach with sodium gluconate to suppress colloid formation [40]. The serum stability results showed a drop of RCP from ~90% at two hours to ~75% by three hours, indicating a narrow window for optimal image acquisition. In vitro receptor binding assay on rat pituitary membrane showed high-affinity, saturable binding (KD ≈ 0.435 nM; RT ≈ 23.2 pmol). In vivo biodistribution of 125I-LHRH in mice showed rapid blood clearance (<1 %ID/g by four hours) with renal uptake (~9.24 %ID/g at 15 min), yielding high target-to-background kinetics for receptor imaging.

Calderon et al. functionalized LHRH with a tridentate Acdien chelator and radiolabeled with ^99m^Tc (^99m^Tc-Acdien-LHRH) [41]. The radiolabeled analogue achieved 99% RCP. In breast cancer cells (MDA-MB-231 cells), the radiolabeled analogue showed time-dependent uptake (15–120 min) that was significantly higher than fac-[^99m^Tc(CO)_3_(H_2_O)_3_]^+^. Excess LHRH competitively reduced uptake, confirming LHRH-receptor–mediated targeting. These findings indicate a co-ligand-free LHRH-carbonyl-Tc design with clean radiochemistry and selective in vitro targeting appropriate for in vivo testing.

Overall, these studies highlight the potential of LHRH derivative peptides radiolabeled with ^111^In or ^99m^Tc. The receptor-targeted imaging approach offers an advantage by increasing the chance of related tumor detection, decreasing false negative and nonspecific accumulations. Nevertheless, ^111^In is burdened by its longer half-life, poorer image quality and less favorable dosimetry and higher costs compared to ^99m^Tc.

In the QUADAS analysis, the main source of bias was related to the unspecified animal origin and to the animal sex distribution.

#### 3.3.2. Radiolabeled LHRH in PET Imaging Applications

In this section all LHRH-derived radiopharmaceuticals useful for PET imaging are mentioned.

In two studies, Zoghi and coworkers reported the design, radiolabeling and preclinical evaluation of two novel ^68^Ga-labeled analogues of Triptorelin, [^68^Ga]-DOTA-Hyd-TRP [42] and [^68^Ga]-DOTA-TRP [43] peptides, useful in PET imaging of GnRH receptor-positive tumors.

In the first study, Zoghi and coworkers [42] optimized synthesis, radiolabeling and quality controls of [^68^Ga]-DOTA-Hyd-TRP. The biodistribution of the radiopharmaceuticals demonstrated high uptake of the tracer in the kidney (In few minutes) and also in testes. Block studies using Triptorelin demonstrated significant specific uptake in GnRH-rich organs.

In a second study, Zoghi and colleagues reported the development and evaluation of [^68^Ga]-DOTA-TRP [43]. The biodistribution of these radiopharmaceuticals demonstrates significant uptake in the kidney, stomach and testes. Significant tumor uptake was observed in 4T1 tumor-bearing female mice 30–120 min post-injection with tumor-to-blood and tumor-to-muscle ratios of 28 and >50 in 1 h, respectively.

In 2008, Schottelius and coworkers described the design, synthesis and preclinical evaluation of new radiolabeled GnRH-I analogues engineered for PET targeting of GnRH-R [44]. Utilizing the D-Lys^6^-GnRH-I peptide, they developed two radiotracers labeled with ^18^F and ^68^Ga. For ^68^Ga-labeling, the peptide was coupled with DOTA on the side chain of D-Lys^6^. To allow ^18^F-labeling via chemo-selective oxime formation, D-Lys^6^-GnRH-I was conjugated with a spacer, like aminohexanoic acid (Ahx) or β-Ala. Three analogues were synthesized based on a D-Lys^6^-GnRH-I scaffold: D-Lys^6^-[^68^Ga]DOTA-GnRH-I, D-Lys^6^-Ahx([^18^F]FBOA)-GnRH-I or D-Lys^6^-β-Ala([^18^F]FBOA)-GnRH-I (FBOA = fluorobenzyloxime acetyl). The ^18^F-labeled Ahx compound showed the most promising results, with high receptor affinity and internalization levels approaching those of the reference ligand [^125^I]Triptorelin. In contrast, the βAla analogue exhibited lower affinity and uptake, while the ^68^Ga-labeled compound showed almost complete loss of binding affinity and ligand internalization. Despite the in vitro success of D-Lys^6^-Ahx([^18^F]FBOA)-GnRH-I, a biodistribution study using [^125^I]Triptorelin in OVCAR-3 tumor-bearing mice revealed poor tumor uptake, with activity in tumors significantly lower than in blood. These findings suggest that the inherently low surface expression of GnRH-R in vivo may limit the utility of these radiopharmaceuticals for imaging applications. In conclusion, while the study identifies D-Lys^6^-Ahx([^18^F]FBOA)-GnRH-I as a high-affinity ligand suitable for receptor targeting, it also highlights the biological limitations of GnRH-R as a viable in vivo imaging target. Further optimization or alternative strategies may be required to overcome the low receptor expression levels observed in tumor models. Ovarian cancer is a highly lethal gynecologic malignancy, often diagnosed at an advanced stage due to the lack of early detection strategies.

Huang et al. synthesized an ^18^F-labeled GnRH agonist by coupling with a small prosthetic group of 4-nitrophenyl-2-[^18^F]fluoropropionate ([^18^F]FP-D-Lys^6^-GnRH) to improve binding affinity and stability [45]. The dynamic micro-PET studies were performed in nude mice bearing PC-3 and SKBR-3 xenografts. The radiolabeled-peptide showed higher tumor uptake in the PC-3 prostate tumor model than in the SKBR-3 breast tumor model. In PC-3-bearing mice, blocking studies indicated that unblocked controls had quicker and more distinct tumor visibility. Co-injection of unlabeled D-Lys^6^-GnRH demonstrated receptor specificity by reducing tumor uptake and contrast (at ~one hour, tumor-to-muscle 3.55 and tumor-to-heart 2.01 in controls versus 1.14 and 0.78 with blocking). The biodistribution showed high accumulation in the gallbladder and abdomen, which restricted the application of this particular ^18^F-labeled GnRH probe in a clinical study.

In 2020, Huang and coworkers redesigned an ^18^F-labeled GnRH probe by incorporating a hydrophilic pegylated linker between the NOTA and a D-Lys^6^-GnRH [46]. For this radiolabeling method the Al^18^F-chelation route enables automated ^18^F labeling with optimal yields and high molar activity (≈35 ± 10% in ~35 min; 20–80 GBq/µmol). Dynamic micro-PET studies were performed in nude mice bearing PC-3 and MDA-MB-231 xenografts. The Al [^18^F]F-NOTA-PEG3-D-Lys^6^-GnRH tracer led to earlier tumor visualization (≤10 min) in PC-3 and MDA-MB-231, with renal clearance and minimal liver uptake, resulting in continuously improved contrast and clearer abdominal backgrounds. Ex vivo micro-PET of dissected rats showed high uptake of radiolabeled peptide in the hypophysis but low uptake in the muscle. These data were consistent with the expression of GnRH receptors in these tissues. Overall, micro-PET investigations in xenograft tumor mouse models revealed considerable tumor uptake and trapping inside tumor tissue in two GnRH-positive models.

As well as for SPECT studies, radiolabeled LHRH-derived peptides were used for PET applications, mainly in breast, prostate and ovarian cancer. D-Lys^6^-GnRH is the most frequently used peptide. ^68^Ga and ^18^F labeled probes showed good results both in vitro and in vivo.

Table 5 provides an overview of the radiolabeled LHRH-derived peptides for PET imaging applications.

In the QUADAS analysis, the main source of bias was related to the unspecified animal origin and to the animal sex distribution. Moreover, sometimes experimental information is not clearly explained.

#### 3.3.3. Nanoradiopharmaceuticals: Applications in Nuclear Medicine Imaging

Nanomaterials have been mainly used as carriers for high drug loads. Recently, they have been labeled with radioisotopes to investigate their pharmacokinetics, pharmacodynamics and in vivo biodistribution [58,59,60]. Nanoradiopharmaceuticals provide several advantages over peptides. They have a high surface area, high radionuclide payload capacity, efficient labeling and control of physicochemical parameters. In addition, they can be used as nano-theranostics by introducing diagnostic or therapeutic isotopes and engineered with ligands for multiple tumor-overexpressed receptors.

Over time, these radiolabeled platforms have shown growing promise for cancer diagnosis and treatment.

Radiolabeled solid-lipid nanocarriers (SLNs) are being developed as theranostic platforms, which combine targeted delivery and nuclear imaging or therapy. They can encapsulate anticancer payloads or receptor-directed ligands, which are subsequently radiolabeled for diagnosis or with therapeutic nuclides for targeted tumor cell apoptosis [61]. De and coworkers conjugated LHRH to doxorubicin (DX)-loaded SLN (FDX-SLN) [47]. Both conjugated and nonconjugated nanoparticles (NPs) were radiolabeled with ^99m^Tc. In PC3-bearing nude mice at three hours post-injection, ^99m^Tc-F-DX-SLN achieved greater tumor accumulation than ^99m^Tc-DX-SLN (7.89 ± 0.38 vs. 6.50 ± 0.19 %ID/g; *p* < 0.05), with a tumor-to-muscle ratio of ~19.4. Both formulations showed significantly high urine excretion (40–47%) and significant hepatic accumulation (22–20%). Planar scintigraphy revealed a strong signal in the xenograft tumor region. However, in mice given DX-SLN, the tumor area was less visible. Overall, LHRH functionalization increased tumor accumulation and imaging contrast without compromising radiochemical stability.

Metal nanoclusters gained attention due to their distinct characteristics, which differ significantly from those of the corresponding atoms and bulk materials. Metal nanoclusters are a promising material for biomedical applications due to their tiny size, biocompatibility, stability, robust processing and luminescence. Gao et al. developed ultrasmall chelator-free radioactive [^64^Cu]Cu nanoclusters using LHRH-conjugated bovine serum albumin (BSA) ([^64^Cu]CuNC@BSA-LHRH) as a scaffold for PET imaging in an orthotopic lung cancer model [48]. Gamma-counting confirmed high renal uptake (>30 %ID/g) and higher tumor uptake with LHRH-targeted nanoclusters (12 %ID/g) compared to non-targeted (3 %ID/g; ~4x) in A549 subcutaneous tumors. In the orthotopic A549 lung model (left lung), the LHRH-targeted nanoclusters provided clear lesion visualization from 0.5 to 4 h post-injection. In vivo near-infrared fluorescence (NIRF) imaging failed to detect the deep orthotopic tumor. However, ex-vivo NIRF and histology revealed preferential tumor/kidney localization and tumor marker CD326 (EpCAM) colocalization for the targeted probe. These results indicated that the limited penetration depth of photons for NIRF imaging hindered non-invasive imaging of deep-seated tumors. Given that most clinical tumors are located in deep positions in the body, PET imaging using [^64^Cu]Cu nanoclusters as tracers would be more appropriate for translating into the clinical setting.

Gold nanoclusters (AuNCs) are gaining attention due to their high fluorescence, nontoxicity, favorable biocompatibility and water solubility. Owing to the remarkable biological features of gold nanoclusters, several synthesis and modification procedures have been widely explored for usage in biomedical applications. Simulations indicate that AuNCs mimic thioredoxin and enhance interactions with target proteins. The peptide may provide AuNCs with additional bioactive properties. Han and coworkers developed ^124^I-labeled LHRH-modified human-serum-albumin-stabilized AuNCs (^124^I-HSALHRH AuNCs) for early diagnosis in a lung cancer model [49]. The nanoconjugate demonstrated excellent in vitro stability (>90% after 24 h) and a modestly prolonged circulation half-life (~1.03 h), compared with BSA-AuNCs (~0.75 h).

Dynamic PET analysis in normal female Sprague Dawley rats showed predominant hepatobiliary clearance (~16.2% ID in the liver at 1 h post-injection), accompanied by gradual thyroid uptake, suggesting partial in vivo de-iodination. These data imply that while the formulation remains stable in circulation, a fraction undergoes metabolic degradation, releasing free iodine. A549 subcutaneous xenografts showed a discernible lesion signal on PET and concordant ex vivo NIRF. At the same time, in an A549 orthotopic lung model, tumors in the left lung were clearly visualized between 2 and 5 h post-injection and exhibited higher radioactivity than the contralateral (non-tumor) lung (*n* = 3, *p* < 0.05). The dual-modality imaging capability provides complementary spatial and functional information for early diagnosis of lung lesions. However, the moderate hepatic accumulation indicates potential off-target exposure, which warrants further optimization. Future strategies such as PEGylation or surface modification could mitigate non-specific hepatic uptake, improve blood circulation and enhance the in vivo safety profile before clinical translation.

Collectively, ^124^I-LHRH-HSA AuNCs exhibited high radiochemical integrity, dual PET/NIRF imaging capability and tumor-specific accumulation, representing a promising platform for the non-invasive detection of deep-seated lung malignancies with further scope for refinement in biocompatibility (Table 6).

In the QUADAS analysis, no potential source of bias was reported for these three studies.

#### 3.3.4. Radiolabeled LHRH for Therapeutic Applications

A novel radiolabeled GnRH receptor targeting peptide was also evaluated for prostate cancer therapy. In particular, the following two studies aim to explore the therapeutic potential of receptor-mediated radiotherapy using a radiolabeled peptide analogue. The same authors labeled the D-Trp^6^-GnRH-I peptide with ^111^In or ^177^Lu.

In 2018, Zoghi and coworkers also reported the design and evaluation of a GnRH-I analogue, conjugated with DOTA and radiolabeled with ^111^In, but as a potential antiproliferative agent for cancer therapy [50]. The radiopharmaceuticals retained high binding affinity for GnRH receptors, which are overexpressed in several tumor types and demonstrated significant cytotoxicity against cancer cell lines in proliferation assays. Preliminary in vivo experiments indicated favorable pharmacokinetics and receptor-mediated tumor uptake, suggesting selective delivery of therapeutic radiation to GnRH receptor–positive tumors. The authors concluded that ^111^In-DOTA-GnRH-I could serve as a promising candidate for targeted radionuclide therapy, combining receptor specificity with direct antiproliferative effects of the peptide analogue.

Moreover, one year later Zoghi and coworkers presented a preclinical study including the development and biological assessment of ^177^Lu-labeled peptide tracer targeting GnRH1 receptors for use in radionuclide therapy of hormone-sensitive tumors [51].

The researchers synthesized a DOTA-conjugated GnRH-I analogue, [^177^Lu]-DOTA-Triptorelin Hydrazide ([^177^Lu]-DOTA-TRPHYD), which enables stable chelation of the therapeutic β-emitting radionuclide ^177^Lu. This radiolabeled peptide presents high stability, suitable for therapeutic use. In vitro evaluation demonstrated that [^177^Lu]-DOTA-TRPHYD retained strong receptor-binding affinity, confirming that neither DOTA conjugation nor radiolabeling compromised the peptide’s ability to recognize GnRH-I receptors, and demonstrated receptor-mediated internalization in GnRH receptor-positive cancer cells. Stability assays showed that the compound remained intact in both PBS and human serum over long periods, supporting its suitability for in vivo use. In vivo biodistribution experiments were conducted in mice bearing GnRH1 receptor-positive tumors (4T1), revealing efficient tumor localization and favorable pharmacokinetics. The tracer exhibited predominantly renal clearance, low background uptake and high tumor-to-nontumor ratios, indicating selective receptor-mediated accumulation in the target tissues. Notably, significant tumor uptake persisted over time, suggesting effective tumor retention of the therapeutic dose. Moreover, the authors carried out a preliminary therapeutic efficacy assessment, observing notable tumor growth inhibition in treated animals compared to controls. These findings suggest the therapeutic potential of [^177^Lu]-DOTA-TRPHYD for targeted treatment of GnRH-expressing tumors. The radiotracer shows promise for theranostic applications, particularly when paired with diagnostic analogues labeled with PET or SPECT radionuclides (^68^Ga or ^111^In) in the same peptide platform. In conclusion, this study introduces ^177^Lu-DOTA-GnRH-I as a promising candidate for targeted radionuclide therapy, combining receptor specificity, favorable in vivo kinetics and preliminary therapeutic efficacy.

All results reported in these two studies provide a strong foundation for further investigations and potential clinical translation of this radiopeptide in the treatment of advanced GnRH receptor-positive cancers, including ovarian carcinoma. Its favorable therapeutic index and receptor specificity also make it a promising candidate for receptor-targeted internal radiotherapy, especially in advanced or metastatic ovarian cancer, where systemic treatment options are limited (Table 7).

In the QUADAS analysis, no potential source of bias was reported for these two studies.

#### 3.3.5. Study of an LHRH-Derived Vaccine by SPECT

In 2007, Chang and colleagues investigated the in vivo biodistribution and pharmacokinetics of a synthetic LHRH vaccine labeled with iodine-131 using longitudinal micro-SPECT/CT imaging in rats [52].

The aim was to noninvasively monitor how the radiolabeled vaccine is retained, distributed and cleared over time, in order to assess its potential as an anticancer immunotherapeutic strategy.

The imaging data revealed prolonged retention of ^131^I-LHRH at the injection site, accumulation in lymphoid tissues and a gradual clearance pattern, which together support its ability to stimulate immune responses.

Pharmacokinetic analysis confirmed these findings, providing a quantitative framework for dosing considerations. Overall, the study demonstrates the utility of micro-SPECT/CT for tracking vaccine dynamics and highlights the therapeutic promise of ^131^I-labeled LHRH vaccines in cancer research (Table 8).

In the QUADAS analysis, generally unclear information is reported, and the insufficiently specified index test and reference standard might have led to the presence of bias in QUADAS analysis.

### 3.4. Final Summary of Results

A table containing all FSH- and LHRH-derivative radiopharmaceuticals identified and explored in this systematic review is reported below (Table 9).

## 4. Conclusions and Future Perspectives

To conclude, our study shows how radiopharmaceuticals targeting LHRH and FSH receptors are an excellent tool for the diagnosis and treatment of tumors such as breast, prostate and ovarian cancers (Table 9).

It is well-known that FSH-R is heterogeneously expressed by a wide range of human and murine cancer cells and in TBVs. Therefore, FSH-R can be considered a good biomarker for primary and metastatic solid cancers, detectable at very early stages of the disease. To this end, radiolabeled monoclonal antibodies and FSHβ-derived peptides were actually studied for PET imaging applications. For our purposes it would appear that FSHβ-derived peptides performances are superior to monoclonal antibodies.

The LHRH receptor can also be considered a great target for cancer detection and personalized treatment. In this case, LHRH derivative peptides for SPECT (^111^In and ^99m^Tc) and PET (^18^F and ^68^Ga) imaging applications and also for therapy were investigated. Currently, applications have been mainly for the diagnosis of breast and prostate cancer. In order to facilitate the functionalization of the peptide with chelating agents, the most used peptide has been the one that sees the replacement of Gly^6^ with a D-Lys^6^, in order to exploit the NH_2_ of the side chain for the functionalization.

Moreover, nanotechnology offers innovative strategies for enhancing the delivery and efficacy of radiopharmaceuticals in nuclear medicine. Gold nanoparticles, owing to their biocompatibility, surface versatility and easy radiolabeling, provide valuable platforms for both diagnostic and therapeutic applications. BSA nanoclusters have recently gained attention as multifunctional nanoplatforms for biomedical applications, including nuclear medicine.

Their intrinsic biocompatibility, low immunogenicity and well-characterized structural properties make them suitable carriers for radionuclides. Likewise, solid-lipid nanocarriers combine stability with controlled release properties, reducing systemic toxicity while enabling targeted delivery. Collectively, these nanostructured systems represent promising tools to advance precision and safety in nuclear medicine.

Overall, all these results suggest that radiolabeled peptides, both standalone and combined with nanotechnology, represent the best compromise. Compared to current commercially available radiopharmaceuticals, the presence of the receptor-selective peptide reduces the possibility of nonspecific uptake (e.g., compared to [^18^F]FDG) and accelerates the biodistribution of the radiopharmaceutical (e.g., compared to ^18^F-PSMA).

Based on the results obtained so far with these radiolabeled compounds, future perspectives may include the chemical modification of peptides to generate more effective radiopharmaceuticals. In addition, novel strategies could involve the replacement of the conventional amino acids with one or more D-amino acids and peptoid monomers within the peptide sequence, in order to stabilize potential enzymatic cleavage sites [62], or adding PEG linkers to extend half-life without compromising receptor binding [46]. Another promising approach might be the development of bifunctional systems combining LHRH and FSH peptides, aimed at enhancing probe uptake in imaging applications or improving therapeutic efficacy [63].

In conclusion, despite the promising applications, none of these radiopharmaceuticals are yet being tested on humans. All candidates remain confined in a pre-clinical setting, probably due to the presence of different biases that limit their impact and reproducibility. The most frequent sources of bias found in selected articles were related to animal selection and reference standards. In order to advance studies and select radiopharmaceuticals for clinical testing, it may be useful to standardize the applied protocols and therefore use common guidelines for those who wish to further study these systems.

## Figures and Tables

**Table 1:** Summary of QUADAS analysis.

First Name and Ref.	Risk of Bias				Applicability Concerns		
	Animal Selection	Index Test	Reference Standard	Flow and Timing	Animal Selection	Index Test	Reference Standard
Radiolabeled biomolecules targeting FSH-R							
Hong [28]	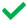	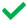	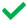	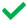	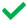	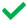	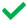
Yang [29]	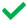	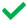	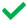	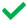	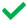	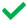	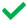
Xu [30]	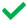	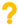	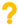	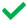	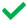	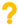	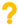
Zhu [31]	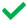	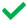	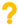	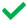	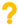	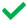	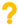
Pan [32]	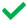	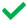	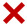	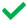	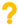	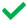	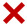
Pan [33]	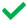	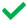	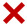	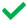	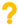	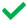	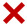
Radiolabeled biomolecules targeting LHRH-R							
*SPECT applications*							
Zoghi [34]	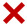	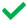	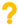	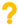	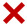	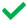	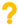
Guo [35]	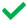	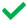	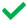	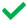	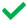	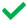	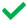
Guo [36]	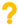	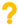	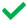	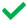	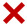	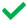	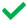
Xu [37]	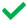	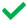	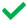	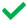	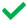	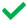	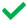
Farahani [38]	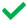	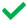	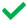	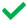	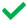	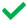	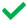
Masteri Farahani [39]	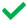	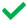	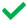	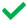	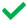	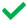	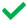
Hao [40]	- **	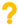	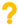	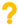	- **	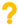	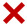
Calderon [41]	- **	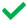	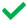	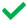	- **	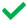	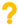
*PET applications*							
Zoghi [42]	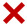	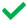	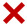	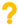	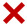	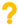	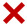
Zoghi [43]	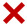	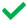	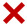	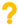	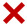	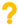	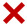
Schottelius [44]	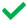	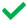	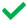	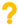	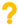	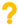	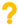
Huang [45]	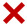	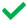	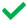	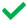	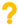	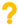	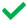
Huang [46]	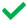	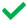	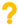	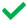	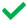	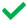	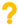
*Nanotechnologies*							
De [47]	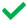	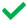	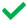	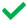	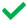	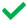	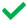
Gao [48]	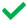	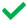	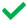	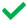	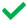	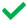	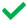
Han [49]	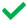	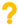	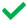	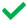	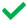	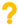	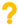
*Therapeutic applications*							
Zoghi [50]	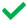	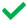	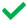	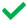	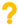	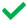	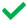
Zoghi [51]	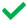	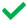	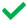	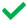	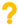	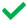	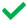
*Other applications*							
Chang [52]	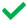	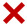	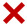	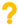	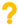	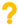	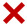

In this table 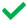 is “low risk of bias”, 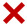 is “high risk of bias”, 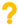 is “unclear risk of bias”; ** missing data because studies are not uniform or missing.

**Table 2:** Characteristics of radiolabeled FSH mAbs useful for PET imaging.

Radiopharmaceutical	mAb	Delivery System	Radionuclide	Chelator	Cancer Model	Ref.
^64^Cu-NOTA-FSH-R-mAb	FSH-R mAb	No drug delivery system	^64^Cu	p-SCN-Bn-NOTA	CAOV-3, SKOV-3, MDA-MB-231, PC-3	[28]
^64^Cu-NOTA-GO-FSH-R-mAb	FSH-R mAb	Nano-graphene oxide (GO) platform	^64^Cu	p-SCN-Bn-NOTA	MDA-MB-231, cbgLuc-MDA-MB-231, SKOV-3	[29]

**Table 3:** Characteristics of radiolabeled FSH-derivative peptides useful for PET imaging.

Radiopharmaceutical	Peptide	Radionuclide	Chelator	RCP %	Cancer Model	Ref.
^18^F-Al-NOTA-MAL-FSH1	FSH1 (FSHβ33–53 *)	^18^F	Al-NOTA-MAL	>98%	PC-3	[30]
^18^F-Al-NOTA-MAL-FSH2	FSH2 (GGGRDN-FSHβ33–53 *)	^18^F	Al-NOTA-MAL	>95%	PC-3	[31]
[^68^Ga]Ga-NOTA-MAL-FSH1	FSH1 (FSHβ33–53 *)	^68^Ga	NOTA-MAL	>95%	PC-3	[32]
[^68^Ga]Ga-NOTA-MAL-FSH4	FSH4 (FSHβ33–53 *-NDRGGG)	^68^Ga	NOTA-MAL	>95%	PC-3	[33]

* FSHβ33–53 peptide sequence is YTRDLVYKDPARPKIQKTCTF.

**Table 4:** Characteristics of radiolabeled LHRH-derivative peptides useful for SPECT imaging.

Radiopharmaceutical	Peptide	Radionuclide	Chelator	RCP%	Cancer Model		Ref.
					In Vitro	In Vivo	
[^111^In]-DOTA-TRP	TRP *	^111^In	DOTA-NCS	>95%	- **	4T1 tumor-bearing female mice	[34]
^111^In-DOTA-Ahx-(D-Lys^6^-GnRH1)	D-Lys^6^-GnRH1	^111^In	DOTA	>95%	MDA-MB-231	MDA-MB-231 cancer-xenografted nude mice	[35]
^111^In-DOTA-Ahx-(D-Lys^6^-GnRH2)	D-Lys^6^-GnRH2						
^111^In-DOTA-Ahx-(L-Lys^6^-GnRH3)	L-Lys^6^-GnRH3						
^111^In-DOTA-Ahx-(D-Lys^6^-GnRH1)	D-Lys^6^-GnRH1	^111^In	DOTA	>95%	- **	DU145 cancer-xenografted nude mice	[36]
^111^In-DOTA-Aoc-D-Phe-(D-Lys^6^-GnRH)	D-Lys^6^-GnRH	^111^In	DOTA	>98%	- **	DU145 cancer-xenografted nude mice	[37]
^111^In-DOTA-βAla-D-Phe-(D-Lys^6^-GnRH)							
^111^In-DOTA-Aun-D-Phe-(D-Lys^6^-GnRH)							
^99m^Tc-HYNIC-GABA-D-Lys^6^-GnRH	D-Lys^6^-GnRH	^99m^Tc	HYNIC	~97%	LN-CaP, DU-145, PC-3	LN-CaP xenografted mice	[38]
^99m^Tc-HYNIC-Ahx-DLys^6^-GnRH	D-Lys^6^-GnRH	^99m^Tc	HYNIC	~97%	LN-CaP, DU-145	LN-CaP xenografted mice	[39]
^99m^Tc-LHRH	GnRH	^99m^Tc	Direct labeling	93.9–96.4%	- **	- **	[40]
^99m^Tc-Acdien-LHRH	D-Lys^6^-GnRH	^99m^Tc	Acdien	>99%	MDA-MB-231	- **	[41]

* TRP: Triptorelin; ** missing data because studies are not uniform or missing.

**Table 5:** Characteristics of radiolabeled LHRH-derivative peptides useful for PET imaging.

Radiopharmaceutical	Peptide	Radionuclide	Chelator	RCP%	Cancer Model		Ref.
					In Vitro	In Vivo	
[^68^Ga]-DOTA-Hyd-TRP	Triptorelin	^68^Ga	pSCN-Bn-DOTA	>99%	- *	4T1 tumor-bearing female mice	[42]
[^68^Ga]-DOTA-TRP	Triptorelin	^68^Ga	pSCN-Bn-DOTA	91–95%	- *	4T1 tumor-bearing female mice	[43]
D-Lys^6^-[^68^Ga]DOTA-GnRH-I	D-Lys^6^-GnRH-I	^68^Ga	DOTA	>99%	EFO-27, SKOV-3, LNCaP, DU-145, MDA-MB-231, SKBR-3	OVCAR-3 tumor-bearing female mice	[44]
D-Lys^6^-Ahx([^18^F]FBOA)-GnRH-I	D-Lys^6^-GnRH-I	^18^F	4-[^18^F] fluorobenzaldehyde	>99%			
D-Lys^6^-β-Ala([^18^F]FBOA)-GnRH-I							
[^18^F]FP-D-Lys^6^-GnRH	D-Lys^6^-GnRH	^18^F	4-nitrophenyl-2-[^18^F]fluoropropionate	>95%	PC-3	PC-3 and SKBR-3 xenografts	[45]
Al [^18^F]F-NOTA-PEG_3_-D-Lys^6^-GnRH	D-Lys^6^-GnRH	^18^F	NOTA + PEG3 linker	≥98%	- *	MDA-MB-231; rat hypophysis (ex vivo)	[46]

* Missing data because studies are not uniform or missing.

**Table 6:** Characteristics of LHRH-R targeted radiolabeled nanotechnologies.

NPs	Peptide	Radionuclide	Chelator/Linker	Physicochemical Characteristics	RCP%	Cancer Model		Ref.
						In Vitro	In Vivo	
SLNPs	LHRH	DX + ^99m^Tc	HYNIC, PEG spacer to DPPE	Size: 245 ± 54 nm (F-DX-SLN); PDI 0.23 ± 0.07; Zeta +33.6 ± 3.4 mV	~92%	PC3 and SKBR3	PC3 xenograft nude mice	[47]
Nanoclusters	LHRH	^64^Cu	Chelator-free [^64^Cu]Cu; LHRH–BSA via EDC/NHS amide	Size: 3.8 ± 0.5 nm (CuNC@BSA-LHRH); Zeta -15.8 mV	97.8%	A549 (LHRH-R^+^) and MRC-5 (LHRH-R^−^)	A549 subcutaneous xenografted tumors	[48]
AuNCs	LHRH	^124^I	Chelator-free radioiodination of Tyr residues using chloramine-T	Size: 6 ± 0.5 nm (LHRH-HSA AuNCs)	98%	- *	A549 xenografted and A549 orthotopic lung cancer model	[49]

* Missing data because studies are not uniform or missing.

**Table 7:** Characteristics of radiolabeled LHRH-derivative peptides useful for cancer therapy.

Radiopharmaceutical	Peptide	Radionuclide	RCP%	Chelator	Cancer Model	Ref.
[^111^In]-DOTA-TRP-HYD	D-Trp^6^-GnRH-I	^111^In	>95%	p-SCN-Bn-DOTA	4T1 tumor-bearing female mice	[50]
[^177^Lu]-DOTA-TRPHYD	D-Trp^6^-GnRH-I	^177^Lu	>98%	p-SCN-Bn-DOTA	4T1 tumor-bearing female mice	[51]

**Table 8:** Characteristics of radiolabeled LHRH-derivative peptides useful as vaccine.

Radiopharmaceutical	Peptide	Radionuclide	RCP%	Ref.
^131^I-labeled LHRH immunogens	LHRH	^131^I	>95%	[52]

**Table 9:** Characteristics of all radiolabeled FSH- and LHRH-derivative peptides.

Radiopharmaceutical	Biomolecule	Cancer	Ref.
Radiolabeled biomolecule targeting FSH-R			
^64^Cu-NOTA-FSH-R-mAb	FSH-R mAb	Ovarian, breast and prostate	[28]
^64^Cu-NOTA-GO-FSH-R-mAb	FSH-R mAb	Ovarian and breast	[29]
^18^F-Al-NOTA-MAL-FSH1	FSH1 (FSHβ33–53)	Prostate	[30]
^18^F-Al-NOTA-MAL-FSH2	FSH2 (GGGRDN-FSHβ33–53)	Prostate	[31]
[^68^Ga]Ga-NOTA-MAL-FSH1	FSH1 (FSHβ33–53)	Prostate	[32]
[^68^Ga]Ga-NOTA-MAL-FSH4	FSH4 (FSHβ33–53-NDRGGG)	Prostate	[33]
Radiolabeled biomolecule targeting LHRH-R			
SPECT applications			
[^111^In]-DOTA-TRP	TRP	Breast	[34]
^111^In-DOTA-Ahx-(D-Lys^6^-GnRH1) ^111^In-DOTA-Ahx-(D-Lys^6^-GnRH2) ^111^In-DOTA-Ahx-(L-Lys^6^-GnRH3)	D-Lys^6^-GnRH1 D-Lys^6^-GnRH2 D-Lys^6^-GnRH3	Breast	[35]
^111^In-DOTA-Ahx-(D-Lys^6^-GnRH1)	D-Lys^6^-GnRH	Prostate	[36]
^111^In-DOTA-Aoc-D-Phe-(D-Lys^6^-GnRH) ^111^In-DOTA-βAla-D-Phe-(D-Lys^6^-GnRH) ^111^In-DOTA-Aun-D-Phe-(D-Lys^6^-GnRH)	D-Lys^6^-GnRH	Prostate	[37]
^99m^Tc-HYNIC-GABA-D-Lys^6^-GnRH	D-Lys^6^-GnRH	Prostate	[38]
^99m^Tc-HYNIC-Ahx-DLys^6^-GnRH	D-Lys^6^-GnRH	Prostate	[39]
^99m^Tc-LHRH	GnRH	-	[40]
^99m^Tc-Acdien-LHRH	D-Lys^6^-GnRH	Breast	[41]
PET applications			
[^68^Ga]-DOTA-Hyd-TRP	Triptorelin	Breast	[42]
[^68^Ga]-DOTA-TRP	Triptorelin	Breast	[43]
D-Lys^6^-[^68^Ga]DOTA-GnRH-I D-Lys^6^-Ahx([^18^F]FBOA)-GnRH-I D-Lys^6^-β-Ala([^18^F]FBOA)-GnRH-I	D-Lys^6^-GnRH-I	Ovarian	[44]
[^18^F]FP-D-Lys^6^-GnRH	D-Lys^6^-GnRH	Prostate and breast	[45]
Al [^18^F]F-NOTA-PEG_3_-D-Lys^6^-GnRH	D-Lys^6^-GnRH	Breast	[46]
Nanotechnologies			
^99m^Tc-DX-SLN	LHRH	Prostate and breast	[47]
[^64^Cu]CuNC@BSA-LHRH	LHRH	Lung	[48]
^124^I-LHRH-HSA AuNCs	LHRH	Lung	[49]
Therapeutic applications			
[^111^In]-DOTA-TRP-HYD	D-Trp^6^-GnRH-I	Breast	[50]
[^177^Lu]-DOTA-TRPHYD	D-Trp^6^-GnRH-I	Breast	[51]
Other applications			
^131^I-labeled LHRH immunogens	LHRH	-	[52]

## Supplementary Materials

The following supporting information can be downloaded at: https://www.mdpi.com/article/10.3390/jcm14217811/s1, Table S1: PRISMA checklist.

## Appendix A

**Table A1:** QUADAS questionnaire for preclinical studies.

Domain	Animal Selection	Index Test	Reference Standard	Flow and Timing
Signaling question (yes, no or unclear)	Does the origin of animals come from company?	(1) Is the origin of bacterial cells certified ATCC? (2) Can the chelator-peptide synthesis be a source of bias? (3) Can the radiolabelling be a source of bias? (4) Were additional in vitro, in vivo, and ex vivo tests performed to support the main results?	Is the reference standard used appropriate for the study?	Is the imaging time appropriated for the study?
Risk of bias (high, low or unclear)	Could the selection of animals have introduced bias?	Could the methodology of experiments have introduced bias?	Could the reference standard or its interpretation have introduced bias?	Could the Radiopharmaceuticals injection administration time be a source of bias?
Concerns about applicability (high, low or unclear)	Are there concerns that the included animals do not match the review questions?	Are there concerns that the index test or its interpretation differ from the review question?	Are there concerns that the target condition as defined by the reference standard does not match the review question?	Could the study flow have introduced bias?
